# Analysis of Genetic Diversity in the American Standardbred Horse Utilizing Short Tandem Repeats and Single Nucleotide Polymorphisms

**DOI:** 10.1093/jhered/esab070

**Published:** 2021-12-10

**Authors:** Elizabeth Esdaile, Felipe Avila, Rebecca R Bellone

**Affiliations:** Veterinary Genetics Laboratory, School of Veterinary Medicine, University of California–Davis, Davis, CA, USA; Veterinary Genetics Laboratory, School of Veterinary Medicine, University of California–Davis, Davis, CA, USA; Veterinary Genetics Laboratory, School of Veterinary Medicine, University of California–Davis, Davis, CA, USA; Department of Population Health and Reproduction, School of Veterinary Medicine, University of California—Davis, Davis, CA, USA

**Keywords:** STRs, SNPs, equine, inbreeding, population genetics

## Abstract

American Standardbreds were developed as a harness racing horse breed. The United States Trotting Association closed the studbook in 1973 and implemented a book size cap in 2009. This study aimed to investigate genetic diversity in the American Standardbred after the studbook cap was introduced using short tandem repeats (STRs) and single-nucleotide polymorphisms (SNPs). Sixteen STRs from horses foaled from 2010 to 2015 and their sires and dams (*n* = 50 621) were utilized to examine allelic richness (*A*_r_), expected heterozygosity (*H*_E_), observed heterozygosity (*H*_O_), unbiased heterozygosity (*H*_U_), inbreeding coefficient (*F*_IS_), and fixation index (*F*_ST_). These analyses found that trotting and pacing sires were less genetically diverse than dams (*H*_E_*P*_*Bonferroni*_ = 0.029 and 6.3 × 10^−5^, respectively) and their offspring (*A*_r_*P*_*Bonferroni*_ = 0.034 and 6.9 × 10^-6^, respectively), and pacing offspring were significantly less diverse than their dams (*H*_E_*P*_*Bonferroni*_ = 2 × 10^-3^). Inbreeding coefficients for trotters (*F*_IS_ = −0.014) and pacers (*F*_IS_ = −0.012) suggest that breeding practices have maintained diversity. Moderate levels of genetic differentiation (0.066 < *F*_ST_ < 0.11) were found between pacing and trotting groups. Additionally, 10 of the most prolific trotting sires and their male offspring (*n* = 84) were genotyped on the 670K Axiom Equine HD Array. *H*_O_ values higher than *H*_E_ (*P* < 0.001), low inbreeding coefficients (mean *F* = −0.064), and mean *F*_ROH_ = 21% indicate relatively high levels of diversity in this cohort, further supporting the STR data. However, in contrast, *H*_O_ values were higher for trotting sires (0.41) than their offspring (0.36). This observation warrants further monitoring of diversity over time. These data provide an updated foundation of diversity indices for further, long-term analysis in the breed.

The American Standardbred was developed during the 19th century as a harness racing horse breed that competes at one of 2 different gaits: the trot (a symmetrical 2-beat, diagonally opposed gait) or the pace (a symmetrical 2-beat, lateral gait). Founding horses of the American Standardbred include individuals of Trotter, Thoroughbred, Norfolk Trotter, Morgan, Barb, Canadian Pacer, and other imported bloodlines (Wallace 1871). Generations of assortative mating separated the American Standardbred into 2 distinct subpopulations based on gait: trotters and pacers. In 1973, the governing body of American Standardbreds, the United States Trotting Association (USTA), closed the studbook (which includes both pacing and trotting horses), restricting registration to offspring of already registered Standardbreds (Adelman 1981; Cothran et al. 1986).

Closing a studbook limits the introduction of new alleles into the gene pool and thus can impact genetic diversity and the health of animals. For example, in Standardbreds, 26% of the 1987 foal crop (6046 foals) were sired by 2% of the sires (60 horses) (King 1992). A continued reduction in diversity has the chance to increase the frequency of genetic disorders. Among those reported for the breed are atrial fibrillation, tarsal osteochondrosis, and recurrent exertional rhabdomyolysis (Lykkjen et al. 2014; Norton et al. 2016; McCoy et al. 2016, 2019; Kraus et al. 2017, 2018).

In addition to reducing genetic diversity, inbreeding has been found to be correlated with reproductive failure in a few horse breeds with high inbreeding levels. For example, in Friesian horses, higher levels of inbreeding were correlated with an increase in the rate of retained placenta (Sevinga et al. 2004). Thus, evaluating genetic diversity is essential to monitor trends within the breed and to develop genomic resources to assist in maintaining diversity and with which to study heritable diseases and reproductive success.

In the 1980s, inbreeding was analyzed in American Standardbreds using pedigree analysis of 14 generations as well as short tandem repeats (STRs). Together, these studies showed higher levels of inbreeding in trotters when compared with pacers (MacCluer et al. 1983; Cothran et al. 1984, 1986; Cothran et al. 1987). A statistically significant association between higher expected heterozygosity and higher foaling and conception rates was also noted in pacers (MacCluer et al. 1983; Cothran et al. 1986). Furthermore, the mean observed total heterozygosity in both trotters and pacers was found to be statistically significantly decreasing over time, more so in trotters than pacers, based on an investigation of over 18 000 trotters and pacers using blood markers (King 1992). The author hypothesized that such a decrease might be attributed to the closed breeding structure and the significant impact that a single popular sire can have on a breed.

Due to the reported decrease in heterozygosity, the closure of the studbook, and a concern for potential health risks due to inbreeding depression, the USTA imposed a studbook limit of 140 mares covered per year for all trotting stallions debuting in 2009. Similarly, a studbook limit of 160 mares covered per year was imposed on pacing sires who debuted in 2009, decreasing to 150 in 2010 and 140 for those who debuted in 2011 or later. It is unknown how studbook restrictions have affected diversity, or if they have mitigated further loss of heterozygosity. To date, the only single-nucleotide polymorphism (SNP)-based study to investigate diversity in Standardbreds utilized data from the 50K SNP array to compare diversity indices of only 15 American Standardbreds (7 trotters and 8 pacers) and 25 Norwegian Standardbreds (trotters), in addition to 35 other breeds. Unlike Norwegian Standardbreds, American Standardbreds were found to have significant excess homozygosity (*F*_IS_), which could be attributed to increased inbreeding in the individuals sampled or substructure based on gait (Petersen et al. 2013).

Given the previous studies from the 1980s, which highlighted differences in inbreeding of trotting and pacing populations when analyzing STRs, excess homozygosity observed in a small sample of American Standardbreds using SNP data, and the concern for the impact of book size on diversity indices, we aim to evaluate genetic diversity in the American Standardbred using both STRs and SNPs after the book size limit was imposed. We also aim to evaluate an extensive data set comprising 50 621 American Standardbreds to further investigate whether pacers are more genetically diverse than trotters (MacCluer et al. 1983). Additionally, our goal was to assess the feasibility of using genotypes from the 670k SNP array on a small subset of trotting individuals as an alternative to the well-established approach of evaluating genetic diversity using STR data. Finally, we hypothesize that dams are maintaining genetic diversity in American Standardbreds.

## Methods

### STR Analysis

#### Data Collection

To establish baseline values of genetic diversity indices to monitor trends over time and the potential effects of the 2009 studbook cap, we evaluated the breeding stock and first 6 foal crops after the rule was imposed (2010–2015). Genotyping records for all foals who’s sires bred for at least 4 consecutive seasons from 2010 to 2015 (“offspring”), as well as their sires (“sires”), and dams (“dams”), were genotyped by Bureau Veritas Laboratories as part of the routine parentage verification process for registration with the USTA and data provided for use in this study. Horses used in this study were categorized by gait phenotype as either trotter or pacer. Phenotyping was performed by the USTA based on performance records.

#### Data Analysis

Horses were grouped according to gait (trotters or pacers) and designated subpopulations (sires, dams, and offspring) (Table 1). Records from 191 pacing dams, 77 trotting dams, 244 pacing offspring, and 40 trotting offspring were removed from the analysis due to duplicate samples and incomplete records, leaving 50 621 horses in our analyses. To compare the impact of book size (i.e., the number of mares bred by a stallion per year) within and between gait types, the sires and offspring were divided into 3 groups based on both the total number of offspring for the years under investigation and sample sizes within each group. Groupings were divided based on approximately 33% of the total offspring and categorized as “high-book,” “mid-book,” and “low-book,” accordingly. The highest producing sires, those that cumulatively sired 40% of trotters and 33% of pacers over the 6 years, were classified as “high-book sires.” Sires that cumulatively sired 28% of trotters and 34% of pacers were classified as “mid-book sires,” and the remaining, lowest producing sires were classified as “low-book sires” (Table 1). Offspring were classified by sire book size in addition to year of birth (Table 1).

**Table 1.** Composition of subpopulations and groups included in the analysis. The percentage is the size of the group when compared with the respective subpopulation (bold). Trotters: high-book sires ranged from an average of 71–107 offspring per year ($\bar{\mu }$ = 78), mid-book sires ranged from an average of 22–67 offspring per year ($\bar{\mu }$ = 37), low-book sires ranged from an average of 0.33–21 offspring per year ($\bar{\mu }$ = 5.9). Pacers: high-book sires ranged from an average of 82–136 offspring per year ($\bar{\mu }$ = 102), mid-book sires ranged from an average of 35–73 offspring per year ($\bar{\mu }$ = 52), low-book sires ranged from an average of 0.67–34 offspring per year ($\bar{\mu }$ = 9.3)

Population			Number of trotters (percent of group type)	Number of pacers (percent of group type)
	Group	Subgroup		
All			22 731	27 890
	**Dams**		**7314**	**9260**
	**Sires**		**171**	**140**
		*High-Book Sires*	13 (*7.6%*)	10 (*7.1%*)
		*Mid-Book Sires*	19 (*11%*)	20 (*14%*)
		*Low-Book Sires*	139 (*81%*)	110 (*79%*)
	**Offspring**		**15 246**	**18 490**
		*Offspring of High-Book Sires*	6105 (*40%*)	6078 (*33%*)
		*Offspring of Mid-Book Sires*	4206 (*28%*)	6259 (*34%*)
		*Offspring of Low-Book Sires*	4935 (*32%*)	6153 (*33%*)
		*2010 Offspring*	2728 (*18%*)	3284 (*18%*)
		*2011 Offspring*	2883 (*19%*)	3497 (*19%*)
		*2012 Offspring*	2814 (*19%*)	3337 (*18%*)
		*2013 Offspring*	2544 *(17%*)	2956 (*16%*)
		*2014 Offspring*	2287 (*15%*)	2886 (*16%*)
		*2015 Offspring*	1990 (*13%*)	2530 (*14%*)

Sixteen STRs were analyzed: AHT4, AHT5, ASB17, ASB2, ASB23, CA425, HMS1, HMS2, HMS3, HMS6, HMS7, HTG10, HTG4, HTG7, LEX33, and VHL20. These markers are routinely used for equine parentage testing and genetic diversity studies as they are located on 12 different chromosomes and are not in linkage disequilibrium (LD) (Sereno et al. 2008; van de Goor et al. 2011; Khanshour et al. 2013). STR data were formatted for analysis with Microsatellite Analyzer 4.05 (MSA) (Dieringer and Schlötterer 2003). For each marker, MSA was used to calculate allelic richness, expected heterozygosity, observed heterozygosity, unbiased heterozygosity, inbreeding coefficient, and pairwise fixation index using 20 000 permutations (*F*_ST_) (Nei 1978; Weir & Cockerham 1984). Mean allelic richness (*A*_r_), mean expected heterozygosity (*H*_E_), mean observed heterozygosity (*H*_O_), mean unbiased heterozygosity (*H*_U_), and mean inbreeding coefficient (*F*_IS_) were then calculated by averaging the values of each locus. *T*-test comparisons of *H*_E_, *H*_O_, *F*_IS_, and *A*_r_, between and within populations, as well as linear models (lm) of offspring foaled from 2010 to 2015, were calculated using R (R Core Team 2020). A Bonferroni-corrected significance level of <0.05 was used to determine the significance of all *t*-tests (*P*_Bonferroni_*=* 1 – (1 − *x*)^*n*^).

### SNP Analysis

#### Data Collection and Genotyping

The total number of foals sired from 2010 to 2015 was used to identify 10 of the most prolific trotting sires from the study period, excluding full-siblings and father-son pairs. These 10 sires contributed to a combined 31% of the total foal crop from 2011 to 2015. To evaluate the representative diversity of these high-book stallions and their male offspring, 6–10 offspring per sire were included in the analysis, based on sample availability, for a total of 96 individuals. Genomic DNA was isolated from mane or tail hair follicles collected at the time of registration using the Qiagen Gentra Puregene Blood Kit as previously described (Mack et al. 2017). Genotyping was conducted at Geneseek Inc. (Lincoln, NE) using the 670K Axiom Equine HD Array. Upon initial quality control, 44 samples had a genotyping rate between 94% and 97%, and 50 samples had a genotyping rate > 97%. Two samples failed initial quality control limits and were removed from downstream analyses.

#### SNP-Based Diversity Analysis

SNP pruning was conducted using PLINK v.1.90 (Purcell et al. 2007; Chang et al. 2015; https://www.cog-genomics.org/plink/1.9/general_usage#cite). Only autosomal SNPs were evaluated. SNPs with minor allele frequency less than 0.05, genotyping rate < 95%, and in strong LD (>0.8) were removed (--maf 0.05 --geno 0.05 --indep-pairwise 50 5 0.8). The pruned data set included 111 688 SNPs. Using the pruned SNP set, expected heterozygosity (*H*_E_), observed heterozygosity (*H*_O_), and method-of-moments inbreeding coefficient estimates (*F*) were calculated using the --het function in PLINK v.1.90 (Purcell et al. 2007). Pairwise comparisons between groups were performed using Mann–Whitney *U*-tests. Principal component analysis (PCA) was also conducted in PLINK v.190 (--pca), and results were plotted using the R package ggplot2 (Wickham 2016). PLINK files were converted to Arlequin format using PGDSpider version 2.1.1.5 (Lischer & Excoffier 2012), and Arlequin version 3.5.2.2 (Excoffier et al. 2007) was used to calculate pairwise *F*_ST_ values between sire families, as well as between sires and their offspring. Significance testing was conducted using 20 000 permutations and ɑ = 0.05.

#### Runs of Homozygosity and Inbreeding Estimates

Genomic inbreeding was also calculated for a subset of 50 individuals with the highest quality SNP data (genotyping rate > 97%), which was comprised of offspring only. Runs of homozygosity (ROH) > 1.0 Mb in length were identified using the --homozyg command in PLINK without prior MAF or LD pruning (Meyermans et al. 2020). A total of 407 091 autosomal SNPs were used for analysis using the following parameters: --homozyg; --homozyg-density 25; --homozyg-gap 1000; --homozyg-kb 1000; --homozyg-snp 30; --homozyg-window-het 1; --homozyg-window-missing 2; --homozyg-window-snp 30. Then, the *F*_ROH_ statistic (McQuillan et al. 2008) was calculated for each individual using the formula: *F*_ROH_ = *L*_ROH_/*L*_AUTO_, where *L*_ROH_ corresponds to the total length of all ROH > 1.0 Mb per animal, and *L*_AUTO_ is the length of the equine autosomal genome (2 474 930 kb) (https://www.ncbi.nlm.nih.gov/genome/145?genome_assembly_id=22878).

## Results

### STR Analysis

#### Allelic Richness (*A*_r_)

In both trotting and pacing subpopulations, high-book sires and mid-book sires had significantly lower A_r_ than low-book sires (trotters *A*_r_ = 4.4 and 5.0; *t*-test: *P*_*Bonferroni*_ = 1.6 × 10^−5^ and 9.3 × 10^−3^; pacers *A*_r_ = 4.0 and 4.7; t-test: *P*_*Bonferroni*_ < 0.001 and 0.017) (Table 2 and Supplementary Table 1). Sires as a whole have lower *A*_r_ when compared with dams and offspring (trotters *A*_r_ = 5.9, 8.1, 8.2; *t*-test: *P*_*Bonferroni*_ = 4.8 × 10^−3^ and 0.034; pacers *A*_r_ = 6.0, 8.4, 8.9; *t*-test: *P*_*Bonferroni*_ = 2.4 × 10^−3^ and 6.9 × 10^−6^). Even when parsed by book size, offspring have higher *A*_r_ than their respective sire groups (*A*_r_—high-book sires and their offspring; trotters = 4.4 and 7.6, pacers = 4.0 and 7.9, mid-book sires and their offspring; trotters = 5.0 and 7.6, pacers = 4.7 and 7.8, low-book sires and their offspring; trotters = 5.9 and 7.4, pacers = 5.9 and 7.9; all *P*_*Bonferroni*_ < 0.02). *A*_r_ of offspring did not differ when parsed by stallion book size or offspring year of birth (*t*-tests: *P*_*Bonferroni*_ > 0.05). *A*_r_ did not differ when comparing respective groups of trotters to pacers, including the comparison of all trotters when compared with all pacers (*t*-test: *P* > 0.05) (Table 2 and Supplementary Table 1).

**Table 2.** Measures of diversity in American Standardbreds calculated using 16 STRs. Presented are allelic richness (*A*_r_), expected, unbiased, and observed heterozygosity (*H*_E_, *H*_U_, and *H*_O_), inbreeding coefficient (*F*_IS_), and pairwise fixation index (*F*_ST_) values of trotting and pacing Standardbreds foaled from 2010 to 2015 and their sires and dams. Statistically significant comparisons are denoted by corresponding letters*.

Population Group * Subgroup*	Trotters					Pacers					Pairwise F__ST__ of Trotters and Pacers
	A_r_	H_E_	H_U_	H_O_	F_IS_	A_r_	H_E_	H_U_	H_O_	F_IS_	
**All**	8.63	0.67	0.66	‡ 0.68	-0.014	9.1	0.67	0.67	‡0.68	-0.012	0.076
Dams	abc 8.06	ab 0.67	ab 0.67	‡, a 0.68	ab -0.015	abc 8.4	abcdefghijk 0.68	abcdefghijkl 0.68	‡, abcdefghi 0.69	-0.017	0.066
Sires	clmnopqrstu 5.94	b 0.65	b 0.66	0.68	-0.041	clmnopqrstu 6.0	a 0.66	a 0.66	0.68	-0.029	0.092
* High Book Sires*	bdefg 4.44	0.64	0.62	0.68	-0.095	bdefg 4.0	0.65	0.64	0.72	-0.11	0.11
* Mid Book Sires*	ahijk 5.00	0.67	0.66	‡ 0.74	a -0.12	ahijk 4.7	0.63	l 0.62	0.65	-0.041	0.076
* Low Book Sires*	abdefghijk 5.88	ac 0.65	ac 0.65	0.67	-0.029	abdefghijk 5.9	c 0.66	c 0.66	0.68	-0.026	0.093
Offspring	gkl 8.19	0.66	0.66	‡ 0.67	-0.015	gkl 8.9	b 0.66	b 0.66	‡, a 0.67	-0.012	0.082
* Offspring of High Book Sires*	dio 7.56	0.65	0.64	‡ 0.67	bcd -0.036	dio 7.9	d 0.66	d 0.65	‡ 0.67	a -0.023	0.099
* Offspring of Mid Book Sires*	ehn 7.63	0.66	0.66	0.67	d -0.011	ehn 7.8	e 0.65	e 0.65	‡, b 0.66	-0.015	0.080
* Offspring of Low Book Sires*	fjm 7.44	0.67	c 0.67	a 0.67	ac -0.0056	fjm 7.9	ac 0.67	ac 0.67	‡, c 0.68	a -0.011	0.077
* 2010 Offspring*	p 7.50	0.66	0.66	0.66	-0.0064	p 7.7	f 0.67	f 0.67	‡, d 0.68	-0.016	0.080
* 2011 Offspring*	q 7.31	0.66	0.66	0.66	-0.0049	q 7.4	g 0.66	g 0.66	‡, e 0.67	-0.014	0.082
* 2012 Offspring*	r 7.13	0.66	0.66	0.67	-0.0052	r 7.9	h 0.66	h 0.66	f 0.67	-0.0089	0.083
* 2013 Offspring*	s 7.25	0.66	0.66	0.66	-0.0041	s 7.5	i 0.66	i 0.66	g 0.67	-0.010	0.081
* 2014 Offspring*	t 7.19	0.66	0.66	0.66	-0.0079	t 7.4	j 0.66	j 0.66	h 0.67	-0.011	0.083
* 2015 Offspring*	u 7.19	0.66	0.66	0.66	-0.0100	u 7.4	k 0.66	k 0.66	‡, i 0.67	-0.012	0.084

*Unique letters in the upper right corner of each cell denote all statistically significant pairwise comparisons within a column based on a t-test and p_*Bonferroni*_ <.05. For example, when looking at A_r_ of trotters, “a” denotes that trotting Dams, Mid Book Sires, and Low Book Sires, are each statistically significantly different in A_r_ from one another. Additionally, “u” in the same column denotes that trotting Sires and 2015 Offspring have statistically significantly different A_r_. ‡ signifies when H_O_ was statistically significantly different from H_E_.

#### Expected Heterozygosity (*H*_E_)

Trotting and pacing dams had higher average *H*_E_ when compared with sires (*H*_E_ trotters = 0.67 and 0.65; H_E_ pacers = 0.68 and 0.66; *t*-test: *P*_*Bonferroni*_ = 0.03 and <0.001, respectively). Pacing dams had statistically significantly higher *H*_E_ than that of offspring (*H*_E_ = 0.68 and 0.66, respectively; *t*-test: *P*_*Bonferroni*_ = 0.002) (Table 2 and Supplementary Table 1). Offspring of low-book sires have significantly higher H_E_ than their sire group (*H*_E_ trotters = 0.67 and 0.65; *H*_E_ pacers = 0.67 and 0.66; *t*-test: trotters, *P*_*Bonferroni*_ = 0.046, pacers *P*_*Bonferroni*_ = 0.024). No significant difference in *H*_E_ was found when comparing trotters and pacers, both as a whole and by group (*t*-test: *P*_*Bonferroni*_ > 0.05) (Table 2 and Supplementary Table 1).

#### Unbiased Heterozygosity (*H*_U_)

Nei’s unbiased heterozygosity (*H*_U_) (Nei 1978) was also calculated when parsing the entire sire population by book size in order to account for differences in the number of individuals among groups. These data corroborate what was identified using *H*_E_, with only one additional statistically significant difference between groups: *H*_U_ of pacing mid-book sires was statistically significantly different than that of dams (*H*_U_ = 0.62 and 0.68, respectively, *t*-test: *P*_*Bonferroni*_ = 0.040) (Table 2 and Supplementary Table 1).

#### Observed Heterozygosity (*H*_O_)

H_O_ of pacing dams was significantly higher than that of offspring as a whole and offspring of mid- and low-book sires (*H*_O_ = 0.69, 0.67, 0.66, and 0.68; *t*-test: *P*_*Bonferroni*_ < 0.001, 3.8 × 10^−3^, 0.012, respectively). In trotters, only *H*_O_ of dams was significantly higher than that of offspring of low-book sires (*H*_O_ = 0.68 and 0.67, respectively; *t*-test: *P*_*Bonferroni*_ = 3.2 × 10^−3^). No other comparisons of *H*_O_ were significantly different (Table 2 and Supplementary Table 1).

#### *H*_O_ and *H*_E_

*H*_O_ was statistically significantly higher than *H*_E_ in both trotting and pacing horses as a whole, dams, total offspring, and offspring of high-book sires. It was also statistically significantly higher in pacing offspring of mid- and low-book sires, pacing offspring foaled in 2010, 2011, and 2015, and trotting mid-book sires (Table 2 and Supplementary Table 1, *t*-test: *P*_*Bonferroni*_ > 0.05).

#### Inbreeding Coefficients (*F*_IS_)

Estimated inbreeding coefficients (*F*_IS_) below zero were observed for all groups (Table 2). In trotters, offspring of high-book sires were found to have lower *F*_IS_ than offspring of mid-book sires, offspring of low-book sires, and dams (*P*_*Bonferroni*_ = 3.3 × 10^−5^, 3.7 × 10^−4^, and 4.5 × 10^−3^, respectively) (Table 2 and Supplementary Table 1). In trotters, dams had higher *F*_IS_ than mid-book sires (*P*_*Bonferroni*_*=* 0.045). In pacers, only offspring of high-book sires had significantly lower *F*_IS_ than offspring of low-book sires (*P*_*Bonferroni*_*=* 0.03). No significant differences in *F*_IS_ were observed when comparing respective trotters and pacers as a whole or by respective groups (t-test: *P* > 0.05) (Supplementary Table 1).

#### Fixation Index (*F*_ST_)

Moderate differentiation (0.05 < *F*_ST_ < 0.15) (Wright 1978) in pairwise *F*_ST_ was found when comparing respective groups of trotters to pacers (Table 1 and Supplementary Table 2). Dams had the lowest pairwise *F*_ST_ between respective groups (*F*_ST_ = 0.066). High-book sires had the highest pairwise *F*_ST_ between respective groups (*F*_ST_ = 0.11), with their offspring not far behind (*F*_ST_ = 0.099). Within the trotting and pacing subpopulations, pairwise *F*_ST_ was below 0.05 in all intergroup comparisons, indicating that little to no genetic differentiation occurred within gait subgroups (Supplementary Table 2).

#### Longitudinal Analysis

**Figure 1.**
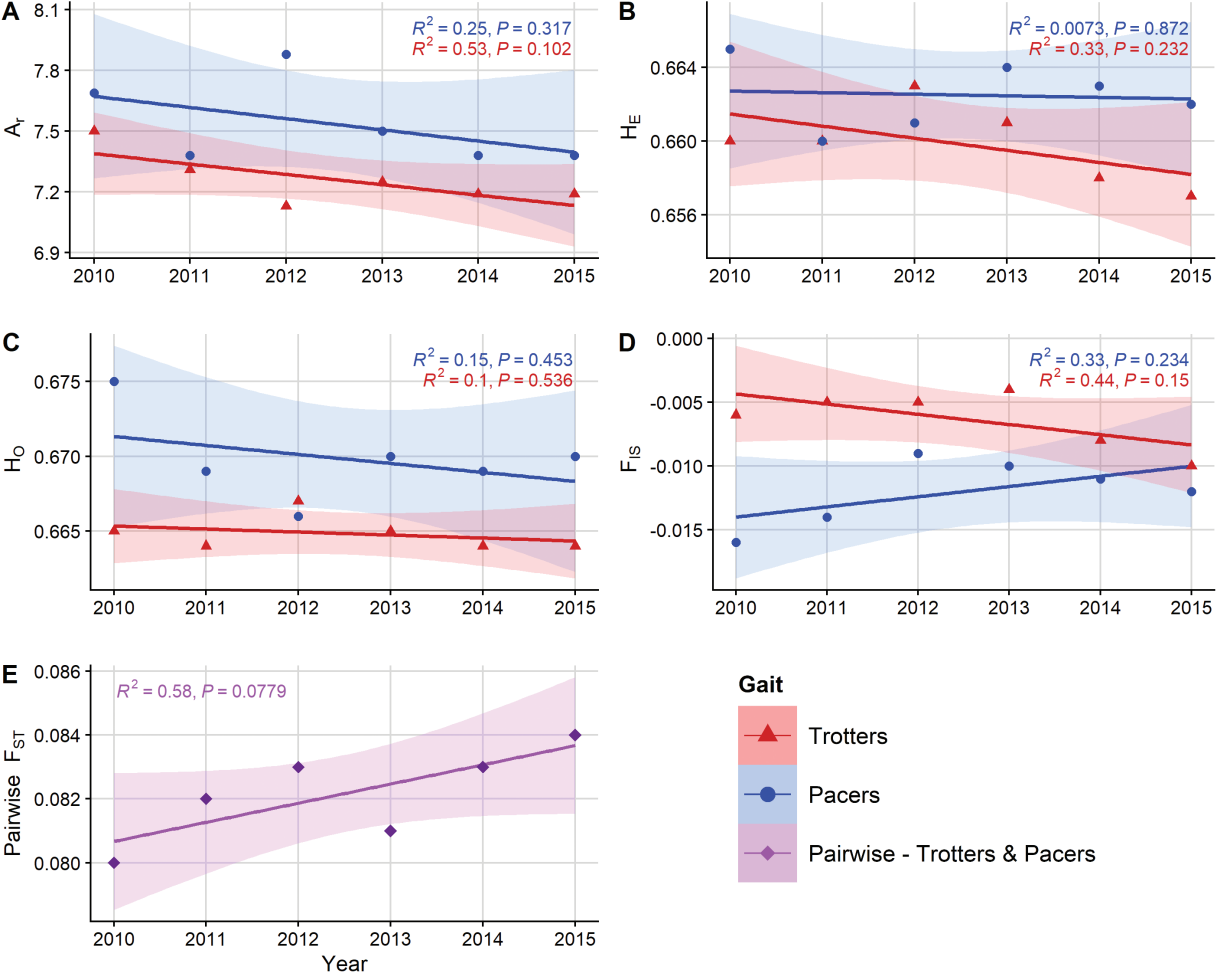
Trends in diversity measures of offspring foaled from 2010 to 2015. *A*_r_, *H*_E_, and *H*_O_ trended downward, suggesting ongoing loss of diversity within the breed. *F*_IS_ differed between groups, increasing in pacers and decreasing in trotters. Pairwise *F*_ST_ trended upwards, suggesting ongoing differentiation between the 2 subpopulations.

Pacing dams were found to have statistically significantly higher *H*_E_ and *H*_O_ than their respective offspring when grouped by year (*P*_*Bonferroni*_ < 0.029), while pacing and trotting sires had lower *A*_r_ than offspring grouped by year (*P*_*Bonferroni*_ < 0.031) (Supplementary Table 2). Additional analysis of offspring parsed by year observed *A*_r_, *H*_E_, and *H*_O_ to be experiencing a downward trend across the 6 years examined (Figure 1). Although still below zero, *F*_IS_ of trotting offspring experienced an overall decrease across the 6 years, but increased in pacing offspring. Across this same period, pairwise *F*_ST_ between offspring of trotters and pacers increased, indicating continued differentiation between the groups. Although there were no statistically significant changes in any of the metrics examined across the 6 years investigated (*P* > 0.05) (Figure 1), these data support the need for further examination of future generations. The different trends in *F*_IS_ of both subpopulations should be explored by SNP analysis to better investigate these trends and potential causes of differentiation.

### SNP Analysis

#### Genomic Diversity

Genome-wide observed heterozygosity (*H*_O_) values ranged from 0.31 to 0.51, with a mean individual heterozygosity of 0.36 (Supplementary Table 3). This value was significantly higher than the expected heterozygosity (*H*_E_) for the study cohort (*P* < 0.001). Additionally, mean observed heterozygosity was significantly higher for sires (0.41) when compared to their offspring (0.36; *P* < 0.001). No significant pairwise differences in heterozygosity levels were detected between half-sibling families.

#### Inbreeding Coefficients (*F*)

Individual inbreeding coefficients (*F*) based on observed and expected heterozygosity showed low estimates of inbreeding and an excess of heterozygosity in the study cohort, ranging from −0.48 to 0.086 (mean = −0.064) (Supplementary Table 3). Sires were found to be significantly less inbred than their offspring, as expected, based on mean *F* (−0.18 and −0.051, respectively; *P* < 0.001), but no significant pairwise differences in inbreeding levels were found between half-sibling families.

#### ROH Identification and *F*_ROH_ Estimation

**Figure 2.**
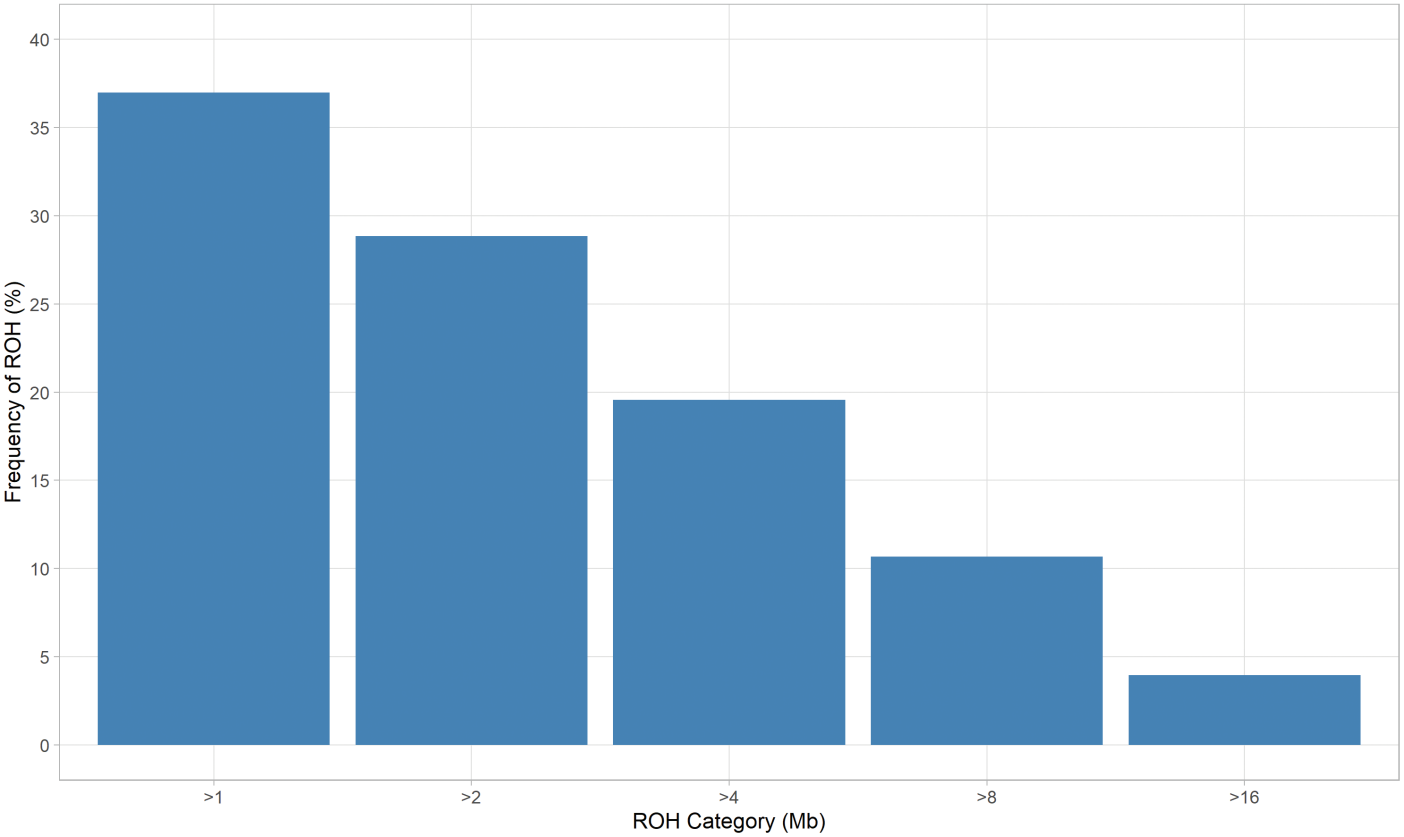
Frequency of runs of homozygosity (ROH) based on length categories (in Mb) identified in the cohort of 50 Standardbreds with highest quality genotyping data.

A total of 5756 ROH above 1 Mb in length were identified in the subset of 50 Standardbreds with high-quality SNP genotyping data. The average number of ROH segments per individual was 115 ± 1.8, with values ranging from 82 to 154. The longest ROH identified for an individual spanned 51 Mb of ECA16, or 58% of the total chromosomal length. The average sum of all ROH lengths per animal was 522 Mb (mean ROH length = 4.5 Mb; median ROH length = 2.7 Mb) (Table 3). The number of segments < 2 Mb in length represented 66% of all ROH, whereas segments greater than 8.0 Mb, indicating recent inbreeding, corresponded to 15% of the total (Figure 2, Supplementary Table 4). Genomic inbreeding estimates using the proportion of the genome covered by ROH (*F*_ROH_) for this cohort ranged from 12% to 27% (mean *F*_ROH_ = 21%) (Table 3).

**Table 3.** Runs of homozygosity (ROH) statistics per individual. Mean (Mb), standard error (SE), standard deviation (SD), min (Mb), max (Mb), *S*_ROH_ (mean genome length covered by ROH), *N*_ROH_ (mean number of ROH), *L*_ROH_ (mean ROH length in Mb), and *F*_ROH_ (inbreeding coefficient) in a subset of 50 Standardbreds with high-quality SNP genotyping data

Total sample	Mean	SE	SD	Minimum	Maximum
*S*_ROH_	522	10	73	307	676
*N*_ROH_	115	1.8	13	82	154
*L*_ROH_	4.6	0.09	0.67	3.2	6.2
*F*_ROH_	0.21	0.004	0.03	0.12	0.27

#### Principal Component Analysis

**Figure 3.**
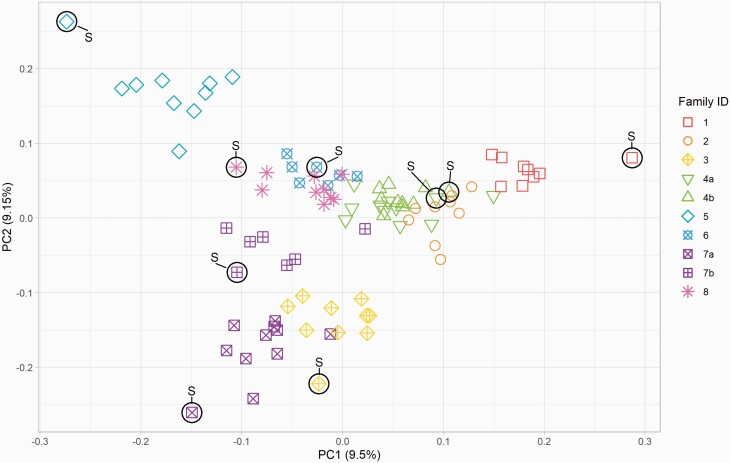
PCA of trotting Standardbreds included in the SNP analysis (*n* = 94). Sire families are denoted by number, and families who share a grandsire are denoted as “a” and “b.” The sire of each family is denoted with an S, except for the sire of family 2, who was excluded due to poor quality data. Numbers in parentheses in the axes titles show the percentage variance explained by each PC.

Relationships among individuals belonging to different sire families were also investigated using PCA (Figure 3). PC 1 and 2 explain 19% of the genetic variation within this sample set. Most families cluster tightly, while some are dispersed across one PC (e.g., family 1) or both PCs (such as family 5). As expected, offspring of half-sibling families that shared a grandsire (indicated by “a” and “b”) grouped in the same cluster of the PCA. The sample set comprising the most likely highest inbred portion of the trotting subpopulation shows a central cluster of 5 families (Family 2, 4a, 4b, 6, 7b, 8, Figure 3).

#### F_ST_

All pairwise *F*_ST_ values calculated between sire families were significant (*P* < 0.001) using 20 000 permutations. Families 4b and 8 showed the lowest level of genetic differentiation (*F*_ST_  =  0.082), whereas the greatest divergence was found between families 6 and 7a (*F*_ST_  = 0.13). As expected, pairwise *F*_ST_ values between families that shared the same grandsire (offspring within families 4a and 4b; 7a and 7b) were among the lowest observed in the dataset (*F*_ST_ = 0.096 for both) (Supplementary Table 5).

## Discussion

In this study, genotyping records of 16 STRs in 50 621 American Standardbreds and genome-wide SNP genotypes from a subset of 94 trotting individuals were used to investigate genetic diversity metrics in the breed across 6 years from 2010 to 2015. Additionally, STRs were utilized to analyze the contribution of dams to within-breed diversity as well as diversity between pacers and trotters. The application of these markers is widespread in population genetic studies due to their robustness to discern genetic differences between individuals using a limited number of highly polymorphic loci and low-quality input DNA. Furthermore, because STR genotypes are readily available for every registered Standardbred, this approach allows for an unbiased investigation of diversity in the entire population in a reproducible and cost-effective way. In contrast, high-density SNP genotyping is rapidly evolving as an alternative strategy to investigate more global genetic diversity, albeit with increased costs.

Analyses utilizing STRs suggested a lack of allelic richness (*A*_r_) in Standardbred sires when compared with offspring and dams, indicating lower diversity in the sire population. When parsed by percentage contribution of offspring, high-book and mid-book sires demonstrated lower *A*_r_ than low-book sires. While it is possible that this difference can be attributed to a smaller number of horses in the high-book sire (*n* = 13 trotters and 10 pacers) and mid-book sire (*n* = 19 trotters and 20 pacers) groups, these may represent true differences in diversity among the groups, and this diversity metric should continue to be monitored to make comparisons across generations.

*H*_E_ of dams was significantly higher than sires and pacing offspring, while offspring as a whole were not significantly different from sires. However, offspring of low-book sires had significantly increased *H*_E_ when compared with their own sire group. A statistically significant difference in *H*_E_ between the offspring of high- and mid-book sires and their respective sire groups was not detected. This may be due to the short time period examined or small group size, which only includes 23 and 39 high- and mid-book sires, respectively.

Although grouping sires based on book size led to small population sizes in the high- and mid-book groups, sires with at least four consecutive breeding seasons during this period were included in the analysis and thus these data represent nearly the entire population from 2010 to 2015. Nei’s unbiased heterozygosity (*H*_U_) was calculated to correct for sample size differences among the groups and results for *H*_U_ mirror that of *H*_E_, with the exception of statistically significantly lower *H*_U_ in pacing mid-book sires compared to pacing dams, which was not detected with *H*_E_. These findings suggest additional data may be necessary to determine the extent of substantial genetic differentiation between the high- and mid-book sire groups compared with dams.

The relatively small number of markers evaluated by the STR analysis could have also hindered the ability to identify small differences in *H*_E_ between sire and offspring groups. In support of this, SNP analyses utilizing genotypes from over 111 000 SNPs in 10 of the most prolific trotting sires and their offspring did detect a difference in genome-wide diversity between these groups. However, contrary to the STR results, the SNP-based analysis found sires to have significantly higher *H*_E_ and *H*_O_ when compared with their offspring. This could be caused by sampling bias in the offspring evaluated and should be validated by testing additional offspring as well as also examining mid-book sires and their offspring with a genome-wide SNP-based analysis.

Significantly higher *H*_O_ when compared with *H*_E_ estimates were detected using both STRs and SNPs, suggesting diversity at the time of book size cap was being maintained in American Standardbreds. Furthermore, other studies comparing SNP-based estimates of heterozygosity to STR results have shown that STRs can yield inflated heterozygosity values as a consequence of ascertainment bias as it pertains to the number of genomic regions under investigation, which is caused by the utilization of a limited number of highly polymorphic markers (Fischer et al. 2017). Overall, our genomic data in trotters support what was observed in the STR data and provide justification for additional analysis using historical STR data with which to make comparisons both before and after the book size cap gaining a broader perspective of diversity in the American Standardbred. Furthermore, high levels of diversity observed by higher values of *H*_O_ when compared with *H*_E_ found in both STR and SNP data are supported by negative inbreeding coefficients, with both metrics indicating that in the years studied, the breeding practices utilized have not negatively impacted diversity in the population.

Additionally, our STR analysis found no significant change in *H*_E_ or any other measured metric across the 6 years of offspring examined. Although, all values except for *F*_IS_ in pacers demonstrate a downward trend, thus showing a potential increase in inbreeding levels. Although not significant, these findings suggest that an increase in the rate of inbreeding may have been detected if a larger timespan was investigated. In support of this, from 2010 to 2015, a decreasing number of sires contributed to an increasing percentage of the annual foal crop. In comparison to a previous report stating that 60 sires (2%) were responsible for 26% of the 1987 foal crop (King 1992), at most 15 sires (4.8% of sires) were responsible for the same 26% of annual foal crop studied, from 2010 to 2015 (13, 15, 15, 14, 12, and 10 sires, respectively). Meanwhile, the top 60 sires were responsible for two-thirds of the offspring studied from 2010 to 2015. This increase in the percent of offspring produced by top sires is concerning because continued utilization of a small sire pool can lead to increased inbreeding practices and therefore leaves the breed vulnerable to recessive genetic diseases that could cause significant economic impact (Adams et al. 2016). It is also possible that a statistically significant difference was not observed in genetic metrics evaluated over the 6-year time frame because the studbook cap of 140 mares per year stabilized any genetic loss that may have occurred in generations prior. Future studies comparing these data to both previous and future generations will help to gain a better understanding of the impact of inbreeding and book size limits within this closed population.

The higher genetic diversity found in dams compared with other groups (as measured by *A*_r_, *H*_O_, *H*_U_, and *H*_E_) may indicate that dams are indeed maintaining the genetic diversity of offspring and counteracting the decreasing number of sires contributing to a larger percentage of the foal crop. If the broodmare population experiences a substantial decrease in the number of individuals or a significant loss of diversity due to shifts in breeding practices, the diversity of the American Standardbred could be threatened.

When comparing subpopulations, the STR analysis found no significant differences in the amount of inbreeding between the trotting and pacing American Standardbreds. This differs from the results of previous pedigree studies, which did support differences in inbreeding of trotters and pacers (MacCluer et al. 1983; Cothran et al. 1984, 1986; ). Nonetheless, the moderate *F*_ST_ values we found indicate that pacing and trotting groups are indeed differentiating from each other, and more so each year. This shows that although the 2 subpopulations have similar levels of genetic diversity, they are 2 moderately different populations. This differentiation was expected as there is minimal interbreeding between trotting and pacing lines, with geneflow most likely to occur from trotters to pacers (Cothran et al. 1987). However, the extent of this geneflow has yet to be investigated. Within-breed diversity indices calculated using SNP data in this study were higher than those previously reported for a cohort of 40 Standardbreds from the USA and Norway (Petersen et al. 2013). In that study, the mean individual inbreeding coefficient (*F*) and expected heterozygosity (*H*_E_) were 0.13 and 0.30, respectively; here, we report a mean *F* of −0.064 and *H*e = 0.34. As demonstrated by these indices, higher diversity levels in our Standardbred cohort might be attributed to greater sample size and SNP density. Furthermore, mean *F*_ROH_ calculated for a subset of 50 Standardbreds in this study (21%) was similar to that found for 55 Thoroughbred horses born in 1994 (*F*_ROH_ = 21%), after which point it increased yearly until 2017 (*F*_ROH_ = 23%) (McGivney et al. 2020). Given that the Thoroughbred is also a closed studbook, it is likely that homozygosity levels in the Standardbred will experience similar increases and loss in diversity over the next 20 years unless carefully monitored and considered in breeding decisions. Furthermore, ROH patterns obtained for this cohort of trotting Standardbreds constitute the foundation for subsequent studies aimed at managing inbreeding rates and identifying genomic signatures of artificial selection for traits of economic importance.

These data constitute a snapshot of diversity trends in American Standardbreds over a 6-year period and illustrate the value in utilizing available STR data to assist in monitoring population-level trends in diversity. Furthermore, the SNP data corroborated STR data from the high-book trotting stallion lines investigated and further illustrates the value in supplementing STR data with high-density SNP data for deeper evaluation. Future SNP-based studies using larger populations of trotting and pacing Standardbreds are required to explore genome-wide diversity in the breed. Together, our data provide a basis of breed-wide genetic diversity in American Standardbreds and serve as a reference point for future studies.

## Supplementary Material

esab070_suppl_Supplementary_Material

## Data Availability

These data will be made available upon reasonable request to the corresponding author.
